# Author Correction: The SARS-CoV-2 spike residues 616/644 and 1138/1169 delineate two antibody epitopes in COVID-19 mRNA COMIRNATY vaccine (Pfizer/BioNTech)

**DOI:** 10.1038/s41598-022-10885-7

**Published:** 2022-04-20

**Authors:** Jessica Andries, Wildriss Viranaicken, Colette Cordonin, Charline Herrscher, Cynthia Planesse, Bénédicte Roquebert, Marie Lagrange-Xelot, Chaker El-Kalamouni, Olivier Meilhac, Patrick Mavingui, David Couret, Gilles Gadea, Philippe Despres

**Affiliations:** 1 Université de La Réunion, INSERM U1187, CNRS UMR 9192, IRD UMR 249, Unité Mixte Processus Infectieux en Milieu Insulaire Tropical (PIMIT), Plateforme Technologique CYROI, 94791 Sainte Clotilde, La Réunion, France; 2 Plate-Forme Technologique CYROI, 94791 Sainte Clotilde, La Réunion, France; 3 Université de La Réunion, INSERM U1188, Unité Mixte Diabète Athérothrombose Thérapies Réunion Océan Indien (DeTROI), Plateforme Technologique CYROI, 94791 Sainte Clotilde, La Réunion, France; 4 Laboratoire CERBA, Parc d’activités « Les Béthunes », 95310 Saint-Ouen-l’Aumône, France; 5 Centre Hospitalier Felix Guyon, Centre Hospitalier Universitaire-La Réunion, 97000 Saint-Denis, La Réunion, France; 6 Groupe Hospitalier Sud Réunion, Centre Hospitalier Universitaire-La Réunion, 97410 Saint-Pierre, La Réunion, France; 7 Present Address: IRCM, U1194, MetaSarc Team, 34298 Montpellier, France

Correction to: *Scientific Reports* 10.1038/s41598-022-10057-7, published online 09 April 2022

The original version of this Article contained a repeated error in the Title, in the Results section under the subheading ‘Antigenic reactivity of synthetic peptides with COMIRNATY vaccine recipient sera’ and in the Methods section under the subheading ‘Serum samples from COMIRNATY vaccine recipients’ where

“COMINARTY”

now reads:

“COMIRNATY”

The original Article has been corrected.

